# (*E*)-1-(2,4-Dinitro­phen­yl)-2-[1-(4-fluoro­phen­yl)ethyl­idene]hydrazine

**DOI:** 10.1107/S1600536812000815

**Published:** 2012-01-14

**Authors:** Hoong-Kun Fun, Boonlerd Nilwanna, Suchada Chantrapromma, Ibrahim Abdul Razak

**Affiliations:** a X-ray Crystallography Unit, School of Physics, Universiti Sains Malaysia, 11800 USM, Penang, Malaysia; b Crystal Materials Research Unit, Department of Chemistry, Faculty of Science, Prince of Songkla University, Hat-Yai, Songkhla 90112, Thailand

## Abstract

The title compound, C_14_H_11_FN_4_O_4_, crystallizes with two essentially planar mol­ecules in the asymmetric unit; the dihedral angles between the benzene rings are 1.57 (15) and 6.17 (15)°. In each mol­ecule, an intra­molecular N—H⋯O hydrogen bond generates an *S*(6) ring. In the crystal, mol­ecules are linked by weak C—H⋯O and C—H⋯F inter­actions into sheets lying parallel to (120). O⋯C [2.980 (4) Å] and O⋯N [2.892 (3) Å] short contacts also occur.

## Related literature

For hydrogen-bond motifs, see: Bernstein *et al.* (1995 ▶). For related structures, see: Chantrapromma *et al.* (2011 ▶); Fun *et al.* (2011 ▶); Nilwanna *et al.* (2011 ▶). For background to the bio­logical activity of hydro­zones, see: Cui *et al.* (2010 ▶). For the stability of the temperature controller used in the data collection, see Cosier & Glazer (1986 ▶). For standard bond lengths, see: Allen *et al.* (1987 ▶).
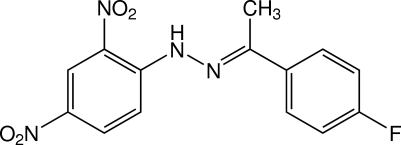


## Experimental

### 

#### Crystal data

C_14_H_11_FN_4_O_4_
*M* *_r_* = 318.27
Triclinic, 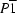

*a* = 8.8278 (3) Å
*b* = 10.9177 (4) Å
*c* = 15.2698 (6) Å
α = 100.649 (3)°
β = 104.948 (3)°
γ = 94.177 (3)°
*V* = 1386.10 (9) Å^3^
*Z* = 4
Mo *K*α radiation
μ = 0.12 mm^−1^
*T* = 100 K
0.32 × 0.30 × 0.04 mm

#### Data collection

Bruker APEX DUO CCD diffractometer
Absorption correction: multi-scan (*SADABS*; Bruker, 2009 ▶) *T*_min_ = 0.962, *T*_max_ = 0.996
19163 measured reflections
5084 independent reflections
3438 reflections with *I* > 2σ(*I*)
*R*_int_ = 0.062

#### Refinement

*R*[*F* ^2^ > 2σ(*F* ^2^)] = 0.066
*wR*(*F* ^2^) = 0.144
*S* = 1.09
5084 reflections
425 parameters
H atoms treated by a mixture of independent and constrained refinement
Δρ_max_ = 0.25 e Å^−3^
Δρ_min_ = −0.34 e Å^−3^

### 

Data collection: *APEX2* (Bruker, 2009 ▶); cell refinement: *SAINT* (Bruker, 2009 ▶); data reduction: *SAINT*; program(s) used to solve structure: *SHELXTL* (Sheldrick, 2008 ▶); program(s) used to refine structure: *SHELXTL*; molecular graphics: *SHELXTL*; software used to prepare material for publication: *SHELXTL* and *PLATON* (Spek, 2009 ▶).

## Supplementary Material

Crystal structure: contains datablock(s) global, I. DOI: 10.1107/S1600536812000815/hb6595sup1.cif

Structure factors: contains datablock(s) I. DOI: 10.1107/S1600536812000815/hb6595Isup2.hkl

Supplementary material file. DOI: 10.1107/S1600536812000815/hb6595Isup3.cml

Additional supplementary materials:  crystallographic information; 3D view; checkCIF report

## Figures and Tables

**Table 1:** Hydrogen-bond geometry (Å, °)

*D*—H⋯*A*	*D*—H	H⋯*A*	*D*⋯*A*	*D*—H⋯*A*
N2*A*—H1*NA*⋯O1*A*	0.87 (3)	1.90 (3)	2.609 (3)	137 (3)
N2*B*—H1*NB*⋯O1*B*	0.84 (3)	2.01 (3)	2.603 (3)	128 (3)
C5*A*—H5*A*⋯O1*B*	0.95	2.48	3.329 (3)	148
C5*B*—H5*B*⋯O1*A*^i^	0.95	2.46	3.253 (3)	141
C6*A*—H6*A*⋯O2*B*	0.95	2.44	3.260 (4)	144
C6*B*—H6*B*⋯O2*A*^i^	0.95	2.44	3.305 (4)	151
C10*A*—H10*A*⋯O4*A*^ii^	0.95	2.53	3.466 (4)	169
C10*B*—H10*B*⋯O4*B*^iii^	0.95	2.43	3.379 (4)	174
C13*B*—H13*B*⋯O2*A*^i^	0.95	2.58	3.487 (4)	159
C14*B*—H14*E*⋯F1*A*^iii^	0.98	2.47	3.205 (4)	131

Symmetry codes: (i) 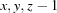; (ii) 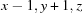; (iii) 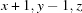.

**Fig. 1.**
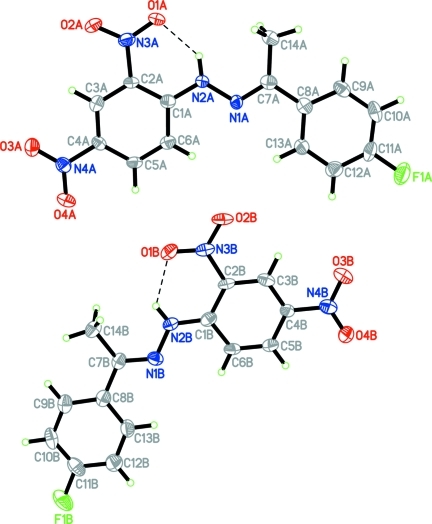
The molecular structure of (I), showing 65% probability displacement ellipsoids. The hydrogen bonds are shown as dashed lines.

**Fig. 2.**
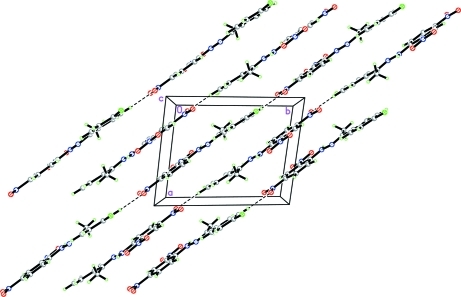
The crystal packing of (I) viewed approximately along the c axis. Hydrogen bonds are shown as dashed lines.

## supplementary crystallographic information

### Comment

Hydrazones are well-known biological compounds with antibacterial, antifungal, antitumor, anti-inflammatory as well as antioxidant properties (e.g. Cui *et al.*, 2010). During the course of our search for antioxidant and antityrosinase compounds, the title compound (I) was synthesized in order to study and compare its biological activity with those of related compounds (Chantrapromma *et al.*, 2011; Fun *et al.*, 2011; Nilwanna *et al.*, 2011). Herein we report the synthesis and crystal structure of (I).

In Fig. 1, there are two crystallographic independent molecules *A* and *B* in the asymmetric unit of (I) with differences in bond angles. The molecular structure of (I), C_14_H_11_FN_4_O_4_ is essentially planar with the the dihedral angle between the 2,4-dinitrophenyl and the 2-fluorophenyl rings being 1.57 (15)° in molecule *A* and 6.17 (15)° in molecule *B*. The central ethylidenehydrazine bridge (N2/N1/C7/C14) is statistically planar with the torsion angles N2–N1–C7–C14 = 0.6 (4) and -0.2 (4)° in molecules *A* and *B*, repectively. The mean plane through this central bridge makes dihedral angles of 3.99 (19) and 4.67 (19)° with the 2,4-dinitrophenyl and 2-fluorophenyl rings, respectively in molecule *A* whereas the corresponding values are 3.20 (19) and 9.19 (19)° in molecule *B*. The two nitro groups of the 2,4-dinitrophenyl unit are almost co-planar with the attached benzene ring with the *r.m.s*. deviation of 0.0083 (3) Å for the twelve non H-atoms, and torsion angles O1–N3–C2–C1 = 0.8 (4)°, O2–N3–C2–C1 = -178.8 (3)°, O3–N4–C4–C3 = 0.7 (4)° and O4–N4–C4–C3 = -180.0 (3)° in molecule *A*; the corresponding values are 0.0258 (3) Å, 3.4 (4), -177.0 (3), 0.2 (4) and -179.0 (3)° in molecule *B*. In each molecule, intramolecular N—H···O hydrogen bond (Fig.1 and Table 1) generates S(6) ring motifs (Bernstein *et al.*, 1995) which help to establish the planarity of the molecules. The bond distances are comparable with the related structures (Chantrapromma *et al.*, 2011; Fun *et al.*, 2011 and Nilwanna *et al.*, 2011).

In the crystal (Fig. 2), the molecules are linked by weak C—H···O and C—H···F interactions (Table 1) into sheets parallel to the (120) plane. O3A···C4A[2.980 (4) Å; symmetry code 1-x, 1-y, 2-z] and O1A···N3B[2.892 (3) Å; symmetry code 1-x, 2-y, 2-z)] short contacts were observed.

### Experimental

The title compound (I) was synthesized by dissolving 2,4-dinitrophenylhydrazine (0.40 g, 2 mmol) in ethanol (10.00 ml) and H_2_SO_4_ (conc.) (98 %, 0.50 ml) was slowly added with stirring. 4-Fluoroacetophenone (0.25 ml, 2 mmol) was then added to the solution with continuous stirring. The solution was stirred for 1 hr yielding an orange solid, which was filtered off and washed with methanol. Orange plates were recrystalized from ethanol by slow evaporation of the solvent at room temperature over several days, Mp. 507-508 K.

### Refinement

Amide H atom was located in a Fourier difference map and refined isotrpically. The remainning H atoms were positioned geometrically and allowed to ride on their parent atoms, with d(C-H) = 0.95 Å for aromatic and 0.98 Å for CH_3_ atoms. The *U*_iso_ values were constrained to be 1.5*U*_eq_ of the carrier atom for methyl H atoms and 1.2*U*_eq_ for the remaining H atoms. A rotating group model was used for the methyl groups.

### Crystal data

**Table:**

C_14_H_11_FN_4_O_4_	*Z* = 4
*M**_r_* = 318.27	*F*(000) = 656
Triclinic, *P*1	*D*_x_ = 1.525 Mg m^−^^3^
Hall symbol: -P 1	Melting point = 507–508 K
*a* = 8.8278 (3) Å	Mo *K*α radiation, λ = 0.71073 Å
*b* = 10.9177 (4) Å	Cell parameters from 5084 reflections
*c* = 15.2698 (6) Å	θ = 1.4–25.5°
α = 100.649 (3)°	µ = 0.12 mm^−^^1^
β = 104.948 (3)°	*T* = 100 K
γ = 94.177 (3)°	Plate, orange
*V* = 1386.10 (9) Å^3^	0.32 × 0.30 × 0.04 mm

### Data collection

**Table:**

Bruker APEX DUO CCD diffractometer	5084 independent reflections
Radiation source: sealed tube	3438 reflections with *I* > 2σ(*I*)
graphite	*R*_int_ = 0.062
φ and ω scans	θ_max_ = 25.5°, θ_min_ = 1.4°
Absorption correction: multi-scan (*SADABS*; Bruker, 2009)	*h* = −10→10
*T*_min_ = 0.962, *T*_max_ = 0.996	*k* = −13→13
19163 measured reflections	*l* = −18→18

### Refinement

**Table:**

Refinement on *F*^2^	Primary atom site location: structure-invariant direct methods
Least-squares matrix: full	Secondary atom site location: difference Fourier map
*R*[*F*^2^ > 2σ(*F*^2^)] = 0.066	Hydrogen site location: inferred from neighbouring sites
*wR*(*F*^2^) = 0.144	H atoms treated by a mixture of independent and constrained refinement
*S* = 1.09	*w* = 1/[σ^2^(*F*_o_^2^) + (0.0581*P*)^2^ + 0.5322*P*] where *P* = (*F*_o_^2^ + 2*F*_c_^2^)/3
5084 reflections	(Δ/σ)_max_ = 0.001
425 parameters	Δρ_max_ = 0.25 e Å^−^^3^
0 restraints	Δρ_min_ = −0.33 e Å^−^^3^

### Special details

**Table:**

Experimental. The crystal was placed in the cold stream of an Oxford Cryosystems Cobra open-flow nitrogen cryostat (Cosier & Glazer, 1986) operating at 100.0 (1) K.
Geometry. All esds (except the esd in the dihedral angle between two l.s. planes) are estimated using the full covariance matrix. The cell esds are taken into account individually in the estimation of esds in distances, angles and torsion angles; correlations between esds in cell parameters are only used when they are defined by crystal symmetry. An approximate (isotropic) treatment of cell esds is used for estimating esds involving l.s. planes.
Refinement. Refinement of F^2^ against ALL reflections. The weighted R-factor wR and goodness of fit S are based on F^2^, conventional R-factors R are based on F, with F set to zero for negative F^2^. The threshold expression of F^2^ > 2sigma(F^2^) is used only for calculating R-factors(gt) etc. and is not relevant to the choice of reflections for refinement. R-factors based on F^2^ are statistically about twice as large as those based on F, and R- factors based on ALL data will be even larger.

### Fractional atomic coordinates and isotropic or equivalent isotropic displacement parameters (Å^2^)

**Table:**

	*x*	*y*	*z*	*U*_iso_*/*U*_eq_	
F1A	−0.1138 (2)	1.38191 (18)	0.72609 (13)	0.0384 (5)	
O1A	0.3270 (2)	0.92455 (19)	1.22388 (13)	0.0232 (5)	
O2A	0.4748 (3)	0.7798 (2)	1.25503 (14)	0.0327 (6)	
O3A	0.6854 (2)	0.54065 (19)	1.03711 (14)	0.0248 (5)	
O4A	0.6408 (3)	0.5739 (2)	0.89707 (14)	0.0302 (6)	
N1A	0.2023 (3)	1.0454 (2)	0.99313 (15)	0.0172 (6)	
N2A	0.2674 (3)	0.9835 (2)	1.06150 (17)	0.0187 (6)	
H1NA	0.265 (4)	0.999 (3)	1.119 (2)	0.046 (11)*	
N3A	0.4076 (3)	0.8423 (2)	1.20111 (16)	0.0217 (6)	
N4A	0.6260 (3)	0.5974 (2)	0.97652 (17)	0.0203 (6)	
C1A	0.3535 (3)	0.8886 (3)	1.04164 (19)	0.0176 (7)	
C2A	0.4241 (3)	0.8195 (3)	1.10755 (18)	0.0168 (7)	
C3A	0.5136 (3)	0.7243 (3)	1.08624 (19)	0.0184 (7)	
H3A	0.5611	0.6795	1.1317	0.022*	
C4A	0.5318 (3)	0.6963 (3)	0.99885 (19)	0.0173 (7)	
C5A	0.4620 (3)	0.7613 (3)	0.93140 (19)	0.0186 (7)	
H5A	0.4753	0.7402	0.8708	0.022*	
C6A	0.3753 (3)	0.8549 (3)	0.95216 (19)	0.0199 (7)	
H6A	0.3284	0.8984	0.9057	0.024*	
C7A	0.1274 (3)	1.1388 (3)	1.01561 (19)	0.0196 (7)	
C8A	0.0606 (3)	1.2029 (3)	0.9396 (2)	0.0199 (7)	
C9A	−0.0301 (3)	1.3018 (3)	0.9510 (2)	0.0233 (7)	
H9A	−0.0515	1.3280	1.0090	0.028*	
C10A	−0.0891 (4)	1.3619 (3)	0.8797 (2)	0.0267 (8)	
H10A	−0.1507	1.4287	0.8879	0.032*	
C11A	−0.0561 (4)	1.3223 (3)	0.7965 (2)	0.0260 (7)	
C12A	0.0323 (3)	1.2265 (3)	0.7816 (2)	0.0256 (7)	
H12A	0.0530	1.2017	0.7233	0.031*	
C13A	0.0904 (4)	1.1669 (3)	0.8530 (2)	0.0225 (7)	
H13A	0.1517	1.1002	0.8435	0.027*	
C14A	0.1104 (4)	1.1822 (3)	1.1112 (2)	0.0260 (8)	
H14A	0.2150	1.1981	1.1563	0.039*	
H14B	0.0443	1.1171	1.1259	0.039*	
H14C	0.0608	1.2598	1.1141	0.039*	
F1B	0.8852 (2)	0.33781 (18)	0.22701 (13)	0.0398 (5)	
O1B	0.4912 (3)	0.8092 (2)	0.72701 (13)	0.0279 (5)	
O2B	0.3390 (3)	0.9487 (2)	0.75796 (13)	0.0313 (6)	
O3B	0.0791 (2)	1.1726 (2)	0.53970 (14)	0.0298 (5)	
O4B	0.0896 (3)	1.1249 (2)	0.39717 (14)	0.0304 (6)	
N1B	0.5742 (3)	0.6777 (2)	0.49060 (16)	0.0207 (6)	
N2B	0.5224 (3)	0.7463 (2)	0.56024 (18)	0.0213 (6)	
H1NB	0.550 (4)	0.730 (3)	0.613 (2)	0.037 (10)*	
N3B	0.3997 (3)	0.8863 (2)	0.70341 (16)	0.0234 (6)	
N4B	0.1260 (3)	1.1111 (2)	0.47797 (17)	0.0241 (6)	
C1B	0.4235 (3)	0.8318 (3)	0.54087 (19)	0.0199 (7)	
C2B	0.3639 (3)	0.9045 (3)	0.60923 (18)	0.0194 (7)	
C3B	0.2684 (3)	0.9967 (3)	0.5887 (2)	0.0208 (7)	
H3B	0.2318	1.0457	0.6354	0.025*	
C4B	0.2279 (3)	1.0158 (3)	0.50028 (19)	0.0186 (7)	
C5B	0.2787 (3)	0.9430 (3)	0.42980 (19)	0.0206 (7)	
H5B	0.2467	0.9561	0.3682	0.025*	
C6B	0.3737 (3)	0.8539 (3)	0.45026 (19)	0.0205 (7)	
H6B	0.4075	0.8051	0.4023	0.025*	
C7B	0.6699 (3)	0.5988 (3)	0.5147 (2)	0.0205 (7)	
C8B	0.7261 (3)	0.5269 (3)	0.4389 (2)	0.0223 (7)	
C9B	0.8137 (4)	0.4275 (3)	0.4514 (2)	0.0252 (7)	
H9B	0.8379	0.4035	0.5096	0.030*	
C10B	0.8665 (4)	0.3627 (3)	0.3798 (2)	0.0281 (8)	
H10B	0.9259	0.2946	0.3885	0.034*	
C11B	0.8308 (4)	0.3995 (3)	0.2966 (2)	0.0279 (8)	
C12B	0.7444 (4)	0.4969 (3)	0.2808 (2)	0.0301 (8)	
H12B	0.7212	0.5202	0.2223	0.036*	
C13B	0.6921 (4)	0.5602 (3)	0.3525 (2)	0.0257 (7)	
H13B	0.6319	0.6276	0.3427	0.031*	
C14B	0.7249 (4)	0.5802 (3)	0.6127 (2)	0.0298 (8)	
H14D	0.7731	0.6609	0.6544	0.045*	
H14E	0.8031	0.5207	0.6164	0.045*	
H14F	0.6345	0.5470	0.6312	0.045*	

### Atomic displacement parameters (Å^2^)

**Table:**

	*U*^11^	*U*^22^	*U*^33^	*U*^12^	*U*^13^	*U*^23^
F1A	0.0397 (12)	0.0405 (12)	0.0411 (12)	0.0156 (10)	0.0087 (9)	0.0230 (10)
O1A	0.0298 (13)	0.0258 (13)	0.0175 (11)	0.0094 (10)	0.0126 (10)	0.0029 (9)
O2A	0.0506 (16)	0.0371 (14)	0.0176 (11)	0.0195 (12)	0.0133 (11)	0.0129 (11)
O3A	0.0231 (12)	0.0232 (12)	0.0298 (12)	0.0056 (10)	0.0068 (10)	0.0096 (10)
O4A	0.0372 (14)	0.0340 (14)	0.0250 (12)	0.0134 (11)	0.0178 (11)	0.0037 (10)
N1A	0.0149 (14)	0.0205 (14)	0.0192 (13)	0.0068 (11)	0.0063 (10)	0.0076 (11)
N2A	0.0236 (15)	0.0207 (15)	0.0161 (13)	0.0090 (12)	0.0095 (11)	0.0063 (11)
N3A	0.0271 (15)	0.0229 (15)	0.0169 (13)	0.0028 (12)	0.0085 (12)	0.0054 (12)
N4A	0.0187 (14)	0.0190 (14)	0.0237 (14)	0.0021 (11)	0.0076 (11)	0.0038 (12)
C1A	0.0160 (16)	0.0187 (17)	0.0193 (15)	−0.0001 (13)	0.0071 (13)	0.0048 (13)
C2A	0.0199 (17)	0.0186 (16)	0.0120 (14)	0.0012 (13)	0.0060 (12)	0.0015 (12)
C3A	0.0144 (16)	0.0181 (17)	0.0208 (16)	−0.0017 (13)	0.0011 (13)	0.0058 (13)
C4A	0.0160 (16)	0.0166 (16)	0.0204 (16)	0.0020 (13)	0.0078 (13)	0.0024 (13)
C5A	0.0211 (17)	0.0215 (17)	0.0157 (15)	0.0023 (14)	0.0094 (13)	0.0042 (13)
C6A	0.0204 (18)	0.0230 (18)	0.0174 (15)	0.0016 (14)	0.0064 (13)	0.0057 (13)
C7A	0.0141 (16)	0.0236 (18)	0.0208 (16)	−0.0004 (14)	0.0061 (13)	0.0030 (13)
C8A	0.0139 (16)	0.0203 (17)	0.0236 (16)	−0.0003 (13)	0.0039 (13)	0.0025 (13)
C9A	0.0217 (18)	0.0205 (18)	0.0293 (17)	0.0019 (14)	0.0125 (14)	0.0018 (14)
C10A	0.0190 (18)	0.0195 (18)	0.042 (2)	0.0068 (14)	0.0085 (15)	0.0057 (15)
C11A	0.0224 (18)	0.0265 (19)	0.0308 (18)	0.0062 (15)	0.0027 (14)	0.0157 (15)
C12A	0.0213 (18)	0.033 (2)	0.0248 (17)	0.0045 (15)	0.0081 (14)	0.0097 (15)
C13A	0.0235 (18)	0.0224 (18)	0.0249 (17)	0.0064 (14)	0.0100 (14)	0.0072 (14)
C14A	0.0309 (19)	0.0259 (19)	0.0244 (17)	0.0087 (15)	0.0131 (15)	0.0037 (14)
F1B	0.0374 (12)	0.0395 (13)	0.0394 (11)	0.0146 (10)	0.0131 (9)	−0.0070 (9)
O1B	0.0352 (14)	0.0316 (14)	0.0193 (11)	0.0074 (11)	0.0068 (10)	0.0108 (10)
O2B	0.0405 (14)	0.0401 (15)	0.0168 (11)	0.0054 (11)	0.0161 (10)	0.0033 (10)
O3B	0.0299 (13)	0.0322 (14)	0.0301 (13)	0.0082 (11)	0.0157 (11)	0.0009 (10)
O4B	0.0368 (14)	0.0355 (14)	0.0229 (12)	0.0156 (11)	0.0084 (10)	0.0115 (10)
N1B	0.0231 (15)	0.0203 (15)	0.0197 (13)	0.0030 (12)	0.0090 (11)	0.0022 (11)
N2B	0.0254 (16)	0.0256 (15)	0.0150 (14)	0.0051 (12)	0.0070 (12)	0.0066 (12)
N3B	0.0272 (16)	0.0280 (16)	0.0153 (13)	−0.0014 (13)	0.0083 (12)	0.0039 (12)
N4B	0.0222 (15)	0.0267 (16)	0.0245 (15)	0.0020 (12)	0.0104 (12)	0.0033 (12)
C1B	0.0191 (17)	0.0210 (17)	0.0194 (16)	−0.0026 (14)	0.0048 (13)	0.0064 (13)
C2B	0.0232 (18)	0.0238 (18)	0.0118 (14)	−0.0012 (14)	0.0071 (13)	0.0037 (13)
C3B	0.0188 (17)	0.0236 (18)	0.0211 (16)	0.0006 (14)	0.0104 (13)	0.0010 (13)
C4B	0.0173 (17)	0.0217 (17)	0.0192 (15)	0.0034 (13)	0.0081 (13)	0.0057 (13)
C5B	0.0218 (18)	0.0250 (18)	0.0154 (15)	0.0016 (14)	0.0061 (13)	0.0042 (13)
C6B	0.0256 (18)	0.0225 (18)	0.0144 (15)	0.0010 (14)	0.0093 (13)	0.0017 (13)
C7B	0.0159 (17)	0.0215 (18)	0.0231 (16)	−0.0005 (14)	0.0016 (13)	0.0084 (14)
C8B	0.0166 (17)	0.0236 (18)	0.0257 (17)	0.0000 (14)	0.0032 (13)	0.0080 (14)
C9B	0.0223 (18)	0.0210 (18)	0.0309 (18)	0.0027 (14)	0.0033 (14)	0.0079 (14)
C10B	0.0176 (18)	0.0217 (18)	0.041 (2)	0.0056 (14)	0.0015 (15)	0.0050 (15)
C11B	0.0212 (18)	0.0248 (19)	0.0331 (19)	0.0023 (15)	0.0072 (15)	−0.0048 (15)
C12B	0.032 (2)	0.032 (2)	0.0258 (17)	0.0075 (16)	0.0073 (15)	0.0064 (15)
C13B	0.0280 (19)	0.0222 (18)	0.0276 (17)	0.0093 (15)	0.0063 (15)	0.0064 (14)
C14B	0.032 (2)	0.037 (2)	0.0239 (17)	0.0096 (16)	0.0063 (15)	0.0136 (15)

### Geometric parameters (Å, °)

**Table:**

F1A—C11A	1.365 (3)	F1B—C11B	1.363 (3)
O1A—N3A	1.241 (3)	O1B—N3B	1.246 (3)
O2A—N3A	1.229 (3)	O2B—N3B	1.232 (3)
O3A—N4A	1.235 (3)	O3B—N4B	1.233 (3)
O4A—N4A	1.235 (3)	O4B—N4B	1.233 (3)
N1A—C7A	1.296 (3)	N1B—C7B	1.286 (4)
N1A—N2A	1.373 (3)	N1B—N2B	1.381 (3)
N2A—C1A	1.364 (3)	N2B—C1B	1.345 (4)
N2A—H1NA	0.87 (4)	N2B—H1NB	0.84 (3)
N3A—C2A	1.451 (3)	N3B—C2B	1.445 (3)
N4A—C4A	1.454 (3)	N4B—C4B	1.455 (4)
C1A—C2A	1.412 (4)	C1B—C6B	1.411 (4)
C1A—C6A	1.414 (4)	C1B—C2B	1.427 (4)
C2A—C3A	1.392 (4)	C2B—C3B	1.388 (4)
C3A—C4A	1.366 (4)	C3B—C4B	1.363 (4)
C3A—H3A	0.9500	C3B—H3B	0.9500
C4A—C5A	1.397 (4)	C4B—C5B	1.405 (4)
C5A—C6A	1.360 (4)	C5B—C6B	1.358 (4)
C5A—H5A	0.9500	C5B—H5B	0.9500
C6A—H6A	0.9500	C6B—H6B	0.9500
C7A—C8A	1.480 (4)	C7B—C8B	1.491 (4)
C7A—C14A	1.499 (4)	C7B—C14B	1.506 (4)
C8A—C9A	1.402 (4)	C8B—C9B	1.391 (4)
C8A—C13A	1.406 (4)	C8B—C13B	1.397 (4)
C9A—C10A	1.382 (4)	C9B—C10B	1.392 (4)
C9A—H9A	0.9500	C9B—H9B	0.9500
C10A—C11A	1.376 (4)	C10B—C11B	1.370 (4)
C10A—H10A	0.9500	C10B—H10B	0.9500
C11A—C12A	1.370 (4)	C11B—C12B	1.374 (4)
C12A—C13A	1.377 (4)	C12B—C13B	1.384 (4)
C12A—H12A	0.9500	C12B—H12B	0.9500
C13A—H13A	0.9500	C13B—H13B	0.9500
C14A—H14A	0.9800	C14B—H14D	0.9800
C14A—H14B	0.9800	C14B—H14E	0.9800
C14A—H14C	0.9800	C14B—H14F	0.9800
			
C7A—N1A—N2A	117.2 (2)	C7B—N1B—N2B	116.2 (2)
C1A—N2A—N1A	118.9 (2)	C1B—N2B—N1B	119.9 (2)
C1A—N2A—H1NA	113 (2)	C1B—N2B—H1NB	122 (2)
N1A—N2A—H1NA	128 (2)	N1B—N2B—H1NB	118 (2)
O2A—N3A—O1A	122.4 (2)	O2B—N3B—O1B	122.2 (2)
O2A—N3A—C2A	118.7 (2)	O2B—N3B—C2B	118.5 (2)
O1A—N3A—C2A	118.8 (2)	O1B—N3B—C2B	119.3 (2)
O3A—N4A—O4A	123.5 (2)	O3B—N4B—O4B	123.3 (3)
O3A—N4A—C4A	118.7 (2)	O3B—N4B—C4B	119.0 (2)
O4A—N4A—C4A	117.8 (2)	O4B—N4B—C4B	117.7 (2)
N2A—C1A—C2A	122.7 (2)	N2B—C1B—C6B	120.8 (3)
N2A—C1A—C6A	120.4 (3)	N2B—C1B—C2B	122.7 (3)
C2A—C1A—C6A	116.9 (3)	C6B—C1B—C2B	116.5 (3)
C3A—C2A—C1A	121.7 (2)	C3B—C2B—C1B	121.6 (3)
C3A—C2A—N3A	115.7 (2)	C3B—C2B—N3B	116.5 (2)
C1A—C2A—N3A	122.5 (2)	C1B—C2B—N3B	121.9 (3)
C4A—C3A—C2A	118.9 (3)	C4B—C3B—C2B	118.9 (3)
C4A—C3A—H3A	120.6	C4B—C3B—H3B	120.6
C2A—C3A—H3A	120.6	C2B—C3B—H3B	120.6
C3A—C4A—C5A	121.1 (3)	C3B—C4B—C5B	121.5 (3)
C3A—C4A—N4A	119.0 (3)	C3B—C4B—N4B	119.2 (3)
C5A—C4A—N4A	119.9 (2)	C5B—C4B—N4B	119.3 (2)
C6A—C5A—C4A	120.2 (3)	C6B—C5B—C4B	119.6 (3)
C6A—C5A—H5A	119.9	C6B—C5B—H5B	120.2
C4A—C5A—H5A	119.9	C4B—C5B—H5B	120.2
C5A—C6A—C1A	121.2 (3)	C5B—C6B—C1B	121.8 (3)
C5A—C6A—H6A	119.4	C5B—C6B—H6B	119.1
C1A—C6A—H6A	119.4	C1B—C6B—H6B	119.1
N1A—C7A—C8A	115.0 (2)	N1B—C7B—C8B	115.4 (3)
N1A—C7A—C14A	123.6 (3)	N1B—C7B—C14B	123.2 (3)
C8A—C7A—C14A	121.4 (3)	C8B—C7B—C14B	121.4 (3)
C9A—C8A—C13A	117.7 (3)	C9B—C8B—C13B	118.4 (3)
C9A—C8A—C7A	122.3 (3)	C9B—C8B—C7B	122.0 (3)
C13A—C8A—C7A	119.9 (3)	C13B—C8B—C7B	119.6 (3)
C10A—C9A—C8A	121.5 (3)	C8B—C9B—C10B	121.0 (3)
C10A—C9A—H9A	119.3	C8B—C9B—H9B	119.5
C8A—C9A—H9A	119.3	C10B—C9B—H9B	119.5
C11A—C10A—C9A	118.1 (3)	C11B—C10B—C9B	118.4 (3)
C11A—C10A—H10A	121.0	C11B—C10B—H10B	120.8
C9A—C10A—H10A	121.0	C9B—C10B—H10B	120.8
F1A—C11A—C12A	118.9 (3)	F1B—C11B—C10B	118.5 (3)
F1A—C11A—C10A	118.2 (3)	F1B—C11B—C12B	118.8 (3)
C12A—C11A—C10A	122.9 (3)	C10B—C11B—C12B	122.7 (3)
C11A—C12A—C13A	118.6 (3)	C11B—C12B—C13B	118.3 (3)
C11A—C12A—H12A	120.7	C11B—C12B—H12B	120.9
C13A—C12A—H12A	120.7	C13B—C12B—H12B	120.9
C12A—C13A—C8A	121.2 (3)	C12B—C13B—C8B	121.2 (3)
C12A—C13A—H13A	119.4	C12B—C13B—H13B	119.4
C8A—C13A—H13A	119.4	C8B—C13B—H13B	119.4
C7A—C14A—H14A	109.5	C7B—C14B—H14D	109.5
C7A—C14A—H14B	109.5	C7B—C14B—H14E	109.5
H14A—C14A—H14B	109.5	H14D—C14B—H14E	109.5
C7A—C14A—H14C	109.5	C7B—C14B—H14F	109.5
H14A—C14A—H14C	109.5	H14D—C14B—H14F	109.5
H14B—C14A—H14C	109.5	H14E—C14B—H14F	109.5
			
C7A—N1A—N2A—C1A	−176.9 (3)	C7B—N1B—N2B—C1B	179.3 (3)
N1A—N2A—C1A—C2A	−179.7 (3)	N1B—N2B—C1B—C6B	−1.1 (4)
N1A—N2A—C1A—C6A	0.1 (4)	N1B—N2B—C1B—C2B	179.1 (3)
N2A—C1A—C2A—C3A	−179.0 (3)	N2B—C1B—C2B—C3B	176.6 (3)
C6A—C1A—C2A—C3A	1.2 (4)	C6B—C1B—C2B—C3B	−3.2 (4)
N2A—C1A—C2A—N3A	1.4 (4)	N2B—C1B—C2B—N3B	−3.3 (4)
C6A—C1A—C2A—N3A	−178.4 (3)	C6B—C1B—C2B—N3B	176.9 (3)
O2A—N3A—C2A—C3A	1.6 (4)	O2B—N3B—C2B—C3B	3.1 (4)
O1A—N3A—C2A—C3A	−178.8 (3)	O1B—N3B—C2B—C3B	−176.5 (3)
O2A—N3A—C2A—C1A	−178.8 (3)	O2B—N3B—C2B—C1B	−177.0 (3)
O1A—N3A—C2A—C1A	0.8 (4)	O1B—N3B—C2B—C1B	3.4 (4)
C1A—C2A—C3A—C4A	−0.7 (4)	C1B—C2B—C3B—C4B	1.6 (4)
N3A—C2A—C3A—C4A	178.9 (3)	N3B—C2B—C3B—C4B	−178.5 (3)
C2A—C3A—C4A—C5A	−0.2 (4)	C2B—C3B—C4B—C5B	1.0 (4)
C2A—C3A—C4A—N4A	179.6 (2)	C2B—C3B—C4B—N4B	179.1 (3)
O3A—N4A—C4A—C3A	0.7 (4)	O3B—N4B—C4B—C3B	0.2 (4)
O4A—N4A—C4A—C3A	−180.0 (3)	O4B—N4B—C4B—C3B	−179.0 (3)
O3A—N4A—C4A—C5A	−179.5 (3)	O3B—N4B—C4B—C5B	178.4 (3)
O4A—N4A—C4A—C5A	−0.1 (4)	O4B—N4B—C4B—C5B	−0.8 (4)
C3A—C4A—C5A—C6A	0.5 (4)	C3B—C4B—C5B—C6B	−1.8 (4)
N4A—C4A—C5A—C6A	−179.3 (3)	N4B—C4B—C5B—C6B	−179.9 (3)
C4A—C5A—C6A—C1A	0.0 (4)	C4B—C5B—C6B—C1B	0.0 (4)
N2A—C1A—C6A—C5A	179.4 (3)	N2B—C1B—C6B—C5B	−177.5 (3)
C2A—C1A—C6A—C5A	−0.8 (4)	C2B—C1B—C6B—C5B	2.4 (4)
N2A—N1A—C7A—C8A	179.5 (2)	N2B—N1B—C7B—C8B	−179.2 (2)
N2A—N1A—C7A—C14A	0.6 (4)	N2B—N1B—C7B—C14B	−0.2 (4)
N1A—C7A—C8A—C9A	176.9 (3)	N1B—C7B—C8B—C9B	−171.8 (3)
C14A—C7A—C8A—C9A	−4.1 (4)	C14B—C7B—C8B—C9B	9.2 (4)
N1A—C7A—C8A—C13A	−4.5 (4)	N1B—C7B—C8B—C13B	8.9 (4)
C14A—C7A—C8A—C13A	174.5 (3)	C14B—C7B—C8B—C13B	−170.2 (3)
C13A—C8A—C9A—C10A	0.2 (4)	C13B—C8B—C9B—C10B	0.1 (4)
C7A—C8A—C9A—C10A	178.8 (3)	C7B—C8B—C9B—C10B	−179.3 (3)
C8A—C9A—C10A—C11A	−0.1 (4)	C8B—C9B—C10B—C11B	0.3 (5)
C9A—C10A—C11A—F1A	−179.8 (3)	C9B—C10B—C11B—F1B	178.7 (3)
C9A—C10A—C11A—C12A	−0.1 (5)	C9B—C10B—C11B—C12B	−0.4 (5)
F1A—C11A—C12A—C13A	179.9 (3)	F1B—C11B—C12B—C13B	−178.9 (3)
C10A—C11A—C12A—C13A	0.2 (5)	C10B—C11B—C12B—C13B	0.2 (5)
C11A—C12A—C13A—C8A	−0.1 (4)	C11B—C12B—C13B—C8B	0.2 (5)
C9A—C8A—C13A—C12A	−0.1 (4)	C9B—C8B—C13B—C12B	−0.4 (5)
C7A—C8A—C13A—C12A	−178.7 (3)	C7B—C8B—C13B—C12B	179.0 (3)

### Hydrogen-bond geometry (Å, °)

**Table:**

*D*—H···*A*	*D*—H	H···*A*	*D*···*A*	*D*—H···*A*
N2A—H1NA···O1A	0.87 (3)	1.90 (3)	2.609 (3)	137 (3)
N2B—H1NB···O1B	0.84 (3)	2.01 (3)	2.603 (3)	128 (3)
C5A—H5A···O1B	0.95	2.48	3.329 (3)	148
C5B—H5B···O1A^i^	0.95	2.46	3.253 (3)	141
C6A—H6A···O2B	0.95	2.44	3.260 (4)	144
C6B—H6B···O2A^i^	0.95	2.44	3.305 (4)	151
C10A—H10A···O4A^ii^	0.95	2.53	3.466 (4)	169
C10B—H10B···O4B^iii^	0.95	2.43	3.379 (4)	174
C13B—H13B···O2A^i^	0.95	2.58	3.487 (4)	159
C14B—H14E···F1A^iii^	0.98	2.47	3.205 (4)	131

Symmetry codes: (i) *x*, *y*, *z*−1; (ii) *x*−1, *y*+1, *z*; (iii) *x*+1, *y*−1, *z*.
